# Large Pleural Effusion Secondary to Primary Signet-Ring Cell Adenocarcinoma of the Lung

**DOI:** 10.7759/cureus.19846

**Published:** 2021-11-23

**Authors:** Sarah Baroud, Sara Moustafa, Halah Ibrahim

**Affiliations:** 1 Internal Medicine, Sheikh Khalifa Medical City, Abu Dhabi, ARE

**Keywords:** left-sided pleural effusion, primary signet ring cell adenocarcinoma of the lung, signet ring cell adenocarcinoma, histopathology (hp), immunohistochemistry staining

## Abstract

Primary signet-ring cell adenocarcinomas (SRCA) that occur outside the gastrointestinal tract are rare with few cases reported in the literature. They carry a poor prognosis and can be a challenge to diagnose. In this case report, we present the case of a 69-year-old female who presented to our hospital with a large left-sided pleural effusion. The findings seen on histopathology and immunohistochemistry (IHC) staining of the pleural fluid were consistent with primary signet-ring cell adenocarcinoma of the lung, and further investigations excluded a secondary source of malignancy.

To the best of our knowledge, this is the first reported case of pleural effusion without an underlying lung mass diagnosed as primary signet-ring cell adenocarcinoma of the lung. Given how rare primary signet-ring cell adenocarcinoma of the lung is, it is important for physicians to carry out a comprehensive diagnostic workup to differentiate between primary and metastatic signet-ring cell adenocarcinoma as this will determine prognosis and treatment.

## Introduction

Signet-ring cell adenocarcinomas (SRCA) are rare tumors that encompass a variety of mucin-producing adenocarcinomas and commonly originate from the gastrointestinal tract [1]. Extragastric signet-ring cell adenocarcinomas are less common and can arise from any other organ as the primary site, such as the urinary bladder, prostate, and breast [2]. Primary signet-ring cell adenocarcinoma of the lung is exceptionally rare, with few cases reported in the literature. It accounts for <1.5% of all lung malignancies and is considered an aggressive tumor with a poor prognosis [3].

## Case presentation

A 69-year-old Indian female presented to our emergency department with a one-month history of progressive shortness of breath associated with orthopnea, nonproductive cough, and chest tightness. Initially, her shortness of breath was mild but progressively worsened during this time, had no exertional component, and was not associated with chest pain. She denied fever, night sweats, cough, or symptoms suggestive of an upper respiratory tract infection. However, she did report decreased appetite and unintentional weight loss of 7 kg over the last month. There was no history of recent travel. The patient was a nonsmoker and did not take any prescription medications, over-the-counter medications, or herbal therapy. She did not have any significant occupational exposures. Past medical history was unremarkable, and surgical history was only notable for a myomectomy in 1994 for endometrial fibroids.

The patient was obese with a body mass index of 30.48 kg/m^2^ (normal reference range: 18.5-24.9 kg/m^2^) and appeared to be in moderate distress. On physical examination, her body temperature was 36.6°C, blood pressure was 162/86 mmHg, heart rate was 74 beats/minute, respiratory rate was 28 breaths/minute, and oxygen saturation on room air was 100%. Examination of the chest demonstrated absent breath sounds, decreased vocal and tactile fremitus, and stony dullness to percussion across the entirety of the left lung. Cardiovascular examination was unremarkable. Abdominal examination revealed an old, healed myomectomy scar at the base of the abdomen; there was no abdominal tenderness, organomegaly, or fluid shift. Breast examination was normal, and there was no cervical, axillary, or inguinal lymphadenopathy. The remaining physical examination was unremarkable.

**Figure 1:**
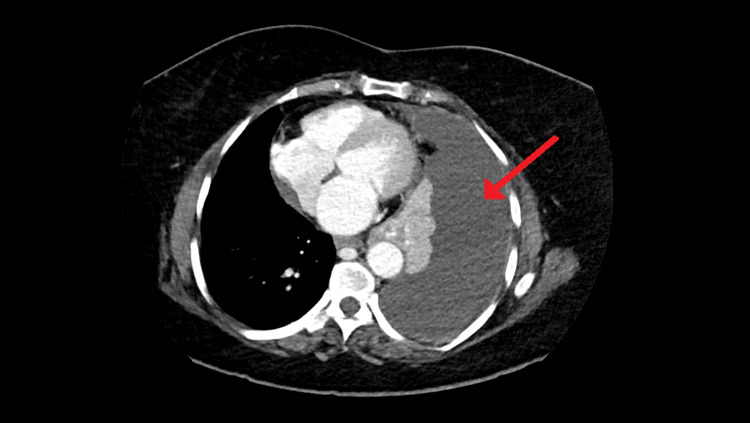
Large left pleural effusion seen on chest computed tomography

Initial laboratory investigations, which included a complete blood count, basic metabolic panel, coagulation profile, lipid panel, liver panel, thyroid panel, and urine analysis, were normal. A COVID-19 point-of-care (POC) test was negative. Her lactate dehydrogenase (LDH) level was 184 IU/L (reference range: 135-214 IU/L), total serum protein was 72 g/L (reference range: 66-87 g/L), and serum glucose was 6.7 mmol/L (reference range: 3.9-7.8 mmol/L). An electrocardiogram (ECG) showed normal sinus rhythm and no ischemic changes. Transthoracic echocardiogram showed grossly normal cardiac function and minimal pericardial effusion. A chest X-ray demonstrated a massive left-sided pleural effusion with underlying lung collapse and mediastinal shift to the right without pneumothorax, right-sided pulmonary consolidation or pleural effusion, or rib destruction. A chest computed tomography (CT) with contrast re-demonstrated the large left pleural effusion with underlying lung collapse and mediastinum shift to the right side. No masses, pleural nodules, and mediastinal or hilar enlargement were visualized. The right lung field was clear (Figure 1).

As the patient was in respiratory distress, a left chest tube was inserted, and a total of 2.5 L of pleural fluid was drained. Pleural fluid analysis was performed, and based on Light's criteria, the findings were consistent with an exudative effusion (Table 1).

**Table 1:** Pleural fluid analysis g: grams; IU: international unit; L: liter; mmol: millimole

Body Fluid Laboratory Markers	Findings
Appearance	Yellow, cloudy, serous fluid
pH	8.0
Protein	50 g/L
Glucose	7.1 mmol/L
Triglyceride	0.23 mmol/L
Lactate dehydrogenase	230 IU/L
Red blood cells	1,000 × 10^6^/L
Nucleated cells	1,067 × 10^6^/L
Polymorphonuclear cells auto %	5.4%
Mononuclear cells auto %	94.6%
Body fluid culture	No growth

Tuberculosis workup with serum QuantiFERON test, sputum mycobacterium tuberculosis GeneXpert PCR, acid-fast bacilli (AFB) smear and cultures, and pleural fluid adenosine deaminase (ADA) level, and autoimmune workup with rheumatoid factor level, antinuclear antibody (ANA), and extractable nuclear antigen (ENA) were sent and returned negative.

**Figure 2:**
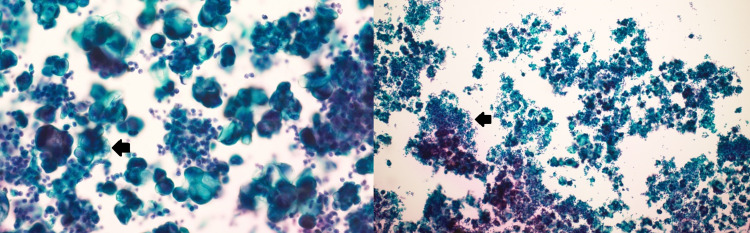
Pleural fluid cytopathology slides showing malignant cells with abundant mutinous cytoplasm

Pleural fluid cytology and cell block demonstrated multiple small clusters of abnormal cells with prominent intracytoplasmic inclusions. Cytopathology examination revealed hypercellular preparations, which included multiple groups of malignant cells, presenting with high nucleocytoplasmic ratios, hyperchromatic chromatin patterns with irregular nuclear contours, and abundant mucinous cytoplasm, characteristic of signet-ring cells. The background demonstrated several mature lymphocytes intermixed with occasional macrophages among blood signifying severe acute inflammation (Figure 2).

**Figure 3:**
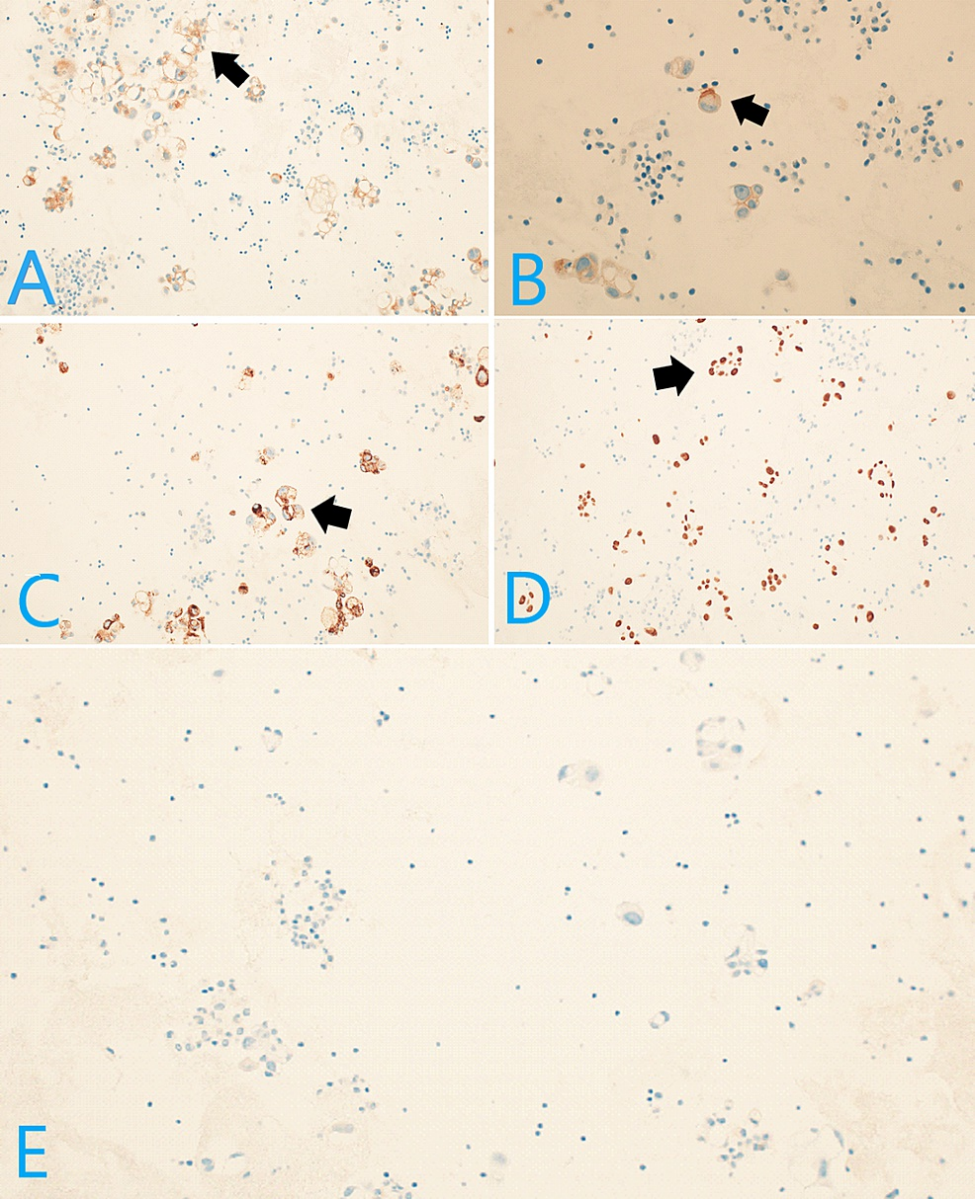
Immunohistochemistry studies demonstrating positivity for MOC31 (A), BerEP4 (weak positive) (B), TTF1 (C), and napsin A (D) and negativity for calretinin (E)

Immunohistochemistry (IHC) staining was positive for MOC31 (Figure 3A), BerEP4 (weak positive) (Figure 3B), cytokeratin 7 (CK7), thyroid transcription factor 1 (TTF1) (Figure 3C), napsin A (Figure 3D), and carcinoembryonic antigen (CEA) and negative for calretinin (Figure 3E), Wilms tumor gene 1 (*WT1*), CK20, estrogen receptor (ER), and progesterone receptor (PR), consistent with adenocarcinoma of primary lung origin.

Based on the findings seen on histopathology and IHC, a provisional diagnosis of primary signet-ring cell adenocarcinoma of the lung was made. However, given the rarity of this condition, further investigations were ordered to exclude secondary cancer. Tumor markers alpha-fetoprotein (AFP), carcinoembryonic antigen (CEA), cancer antigen 125 (CA 125), and carbohydrate antigen 19-9 (CA 19-9) were ordered, and CA 19-9 returned elevated at 126 units/mL (reference range: 0-27 units/mL). Subsequently, a chest, abdomen, and pelvis CT with contrast were done and reported significant improvement of the left-sided pleural effusion post drain insertion, an incidental note of a hypodense thyroid nodule in the right lower lobe, multiple uterine fibroids, a right ovarian cyst measuring 2.4 × 2.7 cm, no obvious bone lesion, and an unremarkable appearance of the liver, gallbladder, spleen, pancreas, adrenals, and kidneys. Thyroid ultrasound showed multiple, small thyroid nodules with no features of malignancy. To further evaluate the ovarian cyst, a pelvis magnetic resonance imaging (MRI) was performed, which reported an atrophic senile fibroid retroverted uterus showing multiple round, small-sized hypovascular, hypointense, and uncomplicated well-encapsulated subserosal, intramural, and submucous fibroids showing no suspicious features, an unremarkable appearance of the cervix and cervical canal, no pelvic collection, a small atrophic left ovary, and a well-encapsulated lobulated right adnexal cystic structure most likely representing a right ovarian cystic lesion, showing no enhancing solid components or papillary projections. Finally, an upper gastrointestinal endoscopy revealed no masses, and a gastric biopsy was negative for any dysplasia or malignancy. An outpatient colonoscopy was scheduled.

Based on the findings seen on histopathology and IHC and no evidence of metastases from other organs, our patient was diagnosed with primary signet-ring cell adenocarcinoma of the lung. The chest tube was removed four days after insertion without complication. She was breathing comfortably and discharged home with outpatient oncology follow-up. The patient did not attend any follow-up appointments. Her condition deteriorated over the following weeks, and she was admitted to an outside hospital with recurrent pleural effusion. She was transferred to the palliative care service and ultimately passed away.

## Discussion

Signet-ring cell adenocarcinomas (SRCA) are rare mucin-producing tumors that can arise from any organ in the body with the stomach, colon, urinary bladder, prostate, and breast being the most common primary sites [1,2]. Primary SRCA of the lung is rare, with few cases reported in the literature. According to the largest series (n = 39) conducted in 2004, lung carcinoma with signet-ring cell components accounted for 1.5% (39/2640) of all lung malignancies, with a mean age of 54.6 years (range: 32-76 years) and nearly equal male/female ratio (1.16:1.00) [3]. Signet-ring cell adenocarcinomas are poorly differentiated tumors and carry a poor prognosis with a five-year survival of 28% [1,3].

Histologically, signet-ring cell carcinoma cells are recognized by their peripherally displaced nuclei secondary to their abundantly mucin-filled cytoplasm [2]. Adenocarcinomas that demonstrate more than 50% signet-ring cell carcinoma cells are labeled as SRCA [3]. In addition, the percentage of visualized signet-ring cell carcinoma cells has been reported to serve as a potential prognostic factor in primary lung tumors, with a higher proportion of signet-ring cells associated with worse outcomes. The diagnosis of primary SRCA of the lung is further made with IHC and radiological imaging [2]. Given how uncommon it is, metastases from other organs should be excluded [3]. IHC, in adjunct with other investigations, has been reported to be a reliable tool in differentiating between primary and metastatic sources of SRCA and in determining the tumor's primary site of origin.

The few available IHC studies published in the literature reported that the combination of a positive CK7, negative CK20, and positive TTF1 was highly specific for pulmonary adenocarcinoma [1]. In one study by Castro et al., 15 cases of primary signet-ring cell adenocarcinoma of the lung were examined; 6/6 tested positive for TTF1 (100%), 9/9 tested positive for CEA (100%), and 3/6 tested positive for CK7 (50%), and all were negative for CK20, estrogen receptor (ER), progesterone receptor (PR), and gross cystic disease fluid protein 15 (GCDFP-15) [4]. In another study by Merchant et al., 17 cases of lung SRCA were investigated and showed that 14/17 cases tested positive for TTF1 (82.4%), and 16/17 demonstrated the CK7+/CK20- patterns (94%) [5]. Our patient tested positive for MOC31, BerEP4 (weak positive), CK7, TTF1, napsin A, and CEA and negative for calretinin, *WT1*, CK20, ER, and PR. The positive CK7 and TTF1 with the negative CK20 is consistent with the diagnosis of primary SRCA of the lung. However, given that these IHC markers may also stain positive in other malignancies, secondary malignancies need to be ruled out. This was done through extensive imaging of the thyroid, chest, abdomen, and pelvis, an upper gastrointestinal endoscopy that revealed no masses, and a gastric biopsy that was negative for any dysplasia or malignancy. Although abdominal and pelvis imaging with a CT scan and MRI did not reveal any colorectal masses, a colonoscopy to definitively rule out a primary colorectal lesion was not performed.

Primary SRCA of the lung is an aggressive tumor with a poor prognosis. This was mainly believed to be a result of its poor response to conventional chemotherapy and radiation therapy treatment over the last two decades. However, recent studies have shown promise with agents that target molecular gene mutations, such as epidermal growth factor receptor(*EGFR*) and anaplastic lymphoma kinase (*ALK*)gene mutations [6]. This is important as about 3%-7% of primary SRCA of the lung are found to be positive for *ALK *gene rearrangement [2]. Crizotinib, a small molecule tyrosine kinase inhibitor, was approved by the Food and Drug Administration in 2011 for the treatment of patients who tested positive for the *ALK* gene rearrangement [7]. Studies thereafter demonstrated its efficacy in patients with non-small cell lung carcinoma (NSCLC) who tested positive for the *ALK *gene rearrangement and*ROS1* receptor tyrosine kinasegene mutation [6,8]; however, there is still not enough data to assess the efficacy of crizotinib in the population with primary SRCA of the lung who test positive for the* ALK* gene mutation [2]. Regardless, it is still recommended that patients diagnosed with SRCA undergo testing for rearrangements in *ALK* genes, as positivity broadens available treatment options [7]. Our patient tested negative for *EGFR*, *ALK*, and* ROS1* gene mutation but positive for *BRAF *gene mutation, which has been demonstrated in 2%-4% of lung adenocarcinomas [9].

## Conclusions

Primary signet-ring cell adenocarcinoma of the lung is a rare and poorly differentiated subtype of adenocarcinoma that creates diagnostic and therapeutic challenges. This case report presents a unique clinical manifestation secondary to primary SRCA of the lung and highlights the importance of pursuing a comprehensive diagnostic workup in order to differentiate between primary and metastatic SRCA and establish appropriate standards of care.
